# Driving a Multistep Electron Transport Chain between Macrocycles with Chemically Mediated Proton-Coupled Electron Transport

**DOI:** 10.1021/acscentsci.4c00459

**Published:** 2024-04-12

**Authors:** Eric H. Hill

**Affiliations:** Institute of Physical Chemistry, University of Hamburg, Grindelallee 117, 20146 Hamburg, Germany; The Hamburg Center for Ultrafast Imaging (CUI), Luruper Chausee 149, 22761 Hamburg, Germany

In this issue of *ACS Central Science*, Sessler, Gong, Henkelman, Bucher, and co-workers report an artificial electron transport chain based on porphyrin analogs and demonstrate chemical control over electron transport pathways within this system via a small molecule mediator, which encouraged proton-coupled electron transport.^1^ Electron transport chains, as found in the cellular respiratory chain and photosynthesis, are ubiquitous and essential in nature, and life would not be possible without them. Proton-coupled electron transport (PCET) describes an electron transfer event which is coupled to the simultaneous transfer of a proton. The term was coined in 1981 to describe the reaction between ruthenium complexes where an electron and proton are transferred simultaneously from Ru^II^–OH_2_^2+^ to Ru^IV^=O^2+^ to give 2 Ru^III^–OH^2+^.^2^ PCET half-reactions involving multiple electrons and protons are ubiquitous in biology. Photosynthesis is a powerful example of PCET, where the transfer of 24 electrons and protons to form carbohydrate from CO_2_ and H_2_O is driven by at least 48 photons, leading to an impressive figure of ≈10^11^ tons of CO_2_ stored annually with ≈10^18^ KJ of energy.^3^ However, in biology, electron transport is generally facilitated by physically separated PCET half reactions, in which minute changes in structure and the chemical environment facilitate forward progression along the chain.^4^

The reactions carried out by the authors of this study focus on the electron transport between two macrocycles, namely cyclo[8]pyrrole and naphthorosarin, and the use of trifluoroacetic acid (TFA) as a mediator species, which was used to favor PCET. In a solution of these two macrocycles, TFA-mediated PCET led to the transfer of an electron from the former to the latter in a single step. Furthermore, the inclusion of an additional redox-active species, I_2_, was used to highlight the high degree of control over PCET across multiple redox steps using TFA. In this case, the electron transport chain led from cyclo[8]pyrrole to I_2_, followed by TFA-mediated PCET from I_3_^–^ to naphthorosarin, resulting in a doubly charged radical species, which then underwent disproportionation along different pathways as controlled by concentration- or TFA-mediated PCET. Overall, this not only served as a first demonstration of PCET occurring between two macrocycles but also established that electron transfer events along an artificial electron transport chain can be regulated by small molecules with a high level of specificity.

**Figure 1:**
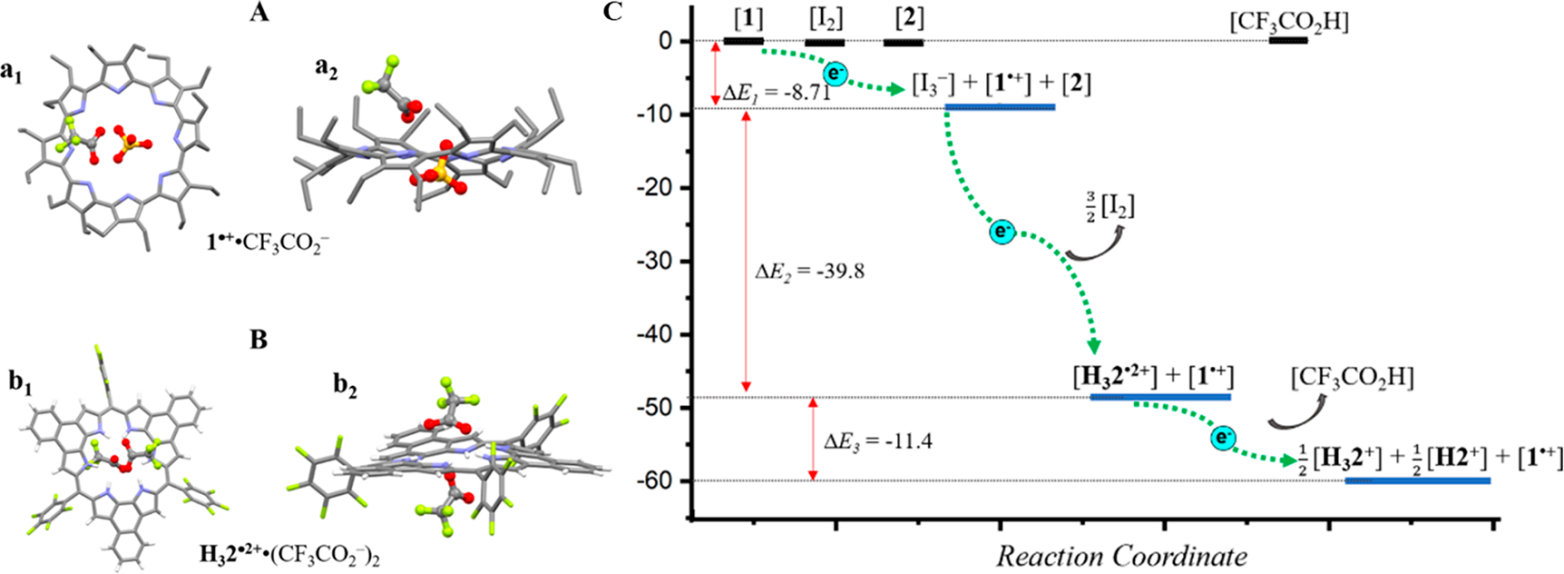
Single crystal complexes of cyclo(8)pyrrole (**1**) and naphthorosarin (**2**): (A) **1**^•+^·CF_3_CO_2_^–^, (B) H_3_**2**^•2+^·(CF_3_CO_2_^–^)_2_; (C) optimized relative-energy profiles for complexes produced along the proposed electron transport chain. Reproduced with permission from ref (1). Copyright 2024 American Chemical Society.^1^

The authors also provide strong support for the thermodynamic control over TFA-mediated PCET via structural characterization. Crystal structures of the radical cations of both macrocycles with TFA were obtained from single crystals, revealing complexes with TFA that support that electron transfer from one macrocycle to the other is coordinated with protonation by TFA (Figure 1a,b). Electronic structure calculations based on the obtained crystal structures provided further support of the interaction of the different components to form an electron transport chain. The reaction energies of the relevant species decreased going down the electron transport chain as proposed by the authors, supporting a thermodynamically driven process (Figure 1c).

**Figure 2:**
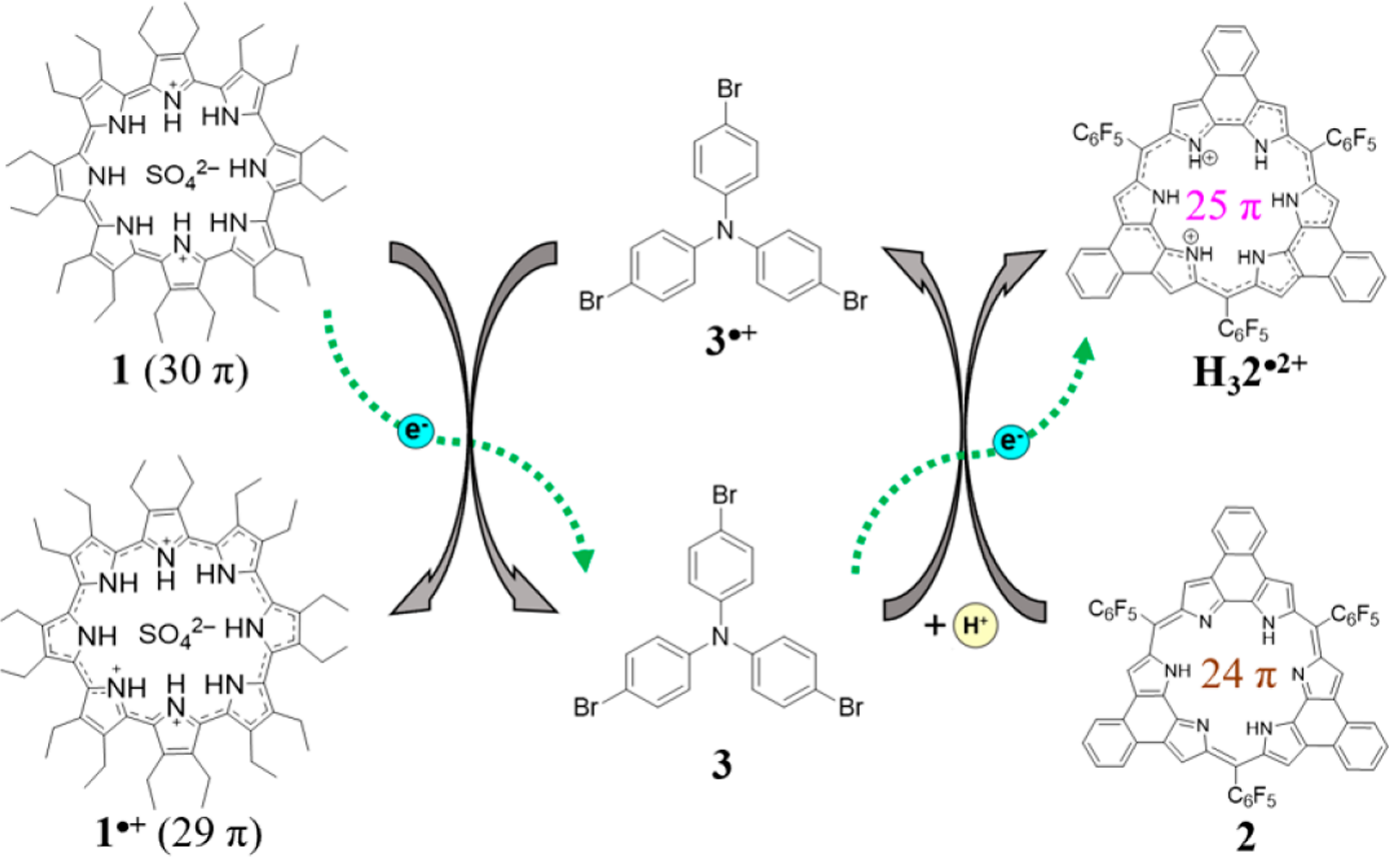
Preliminary results supporting the generalizable strategy: Schematic representation of electron transfer from **1** to **3**^•+^ ([(*p*-BrC_6_H_4_)_3_N^•^]^+^[SbCl_6_]^−^) and further to give H_3_**2**^•2+^. Reproduced with permission from ref (1). Copyright 2024 American Chemical Society.^1^

But how generalizable is this process? Considering that there are four components involved in this electron transport chain, a significant effort is needed to replicate this level of control in a completely different system. However, the authors have already tackled a critical aspect of generalization by showing in the Supporting Information that I_2_ could be replaced with [(*p*-BrC_6_H_4_)_3_N^•^]^+^[SbCl_6_]^−^ (Figure 2).

The level of specificity and control over the electron transport pathway is notable considering that there are three redox-active components, and the authors’ preliminary results (Figure 2) make a strong case for the broader generalizability of their findings. I mirror the authors’ views that these findings should advance the design of artificial electron transport systems for applications across many fields, with potentially great benefit to energy production (including production of solar fuels and thermoelectric energy), conversion, and storage, which are in great need of improvement. In order to tackle challenges in these areas, efforts should be undertaken to couple such chemically mediated PCET approaches with photoredox catalysts,^5^ solid state catalysts,^6^ and photoexcited redox-active species.^7^ Such efforts will provide novel artificial electron transport chains capable of limiting charge recombination in photocatalysts, increasing the efficiency of solar fuel production and harnessing excited-state PCET reactivity that cannot be realized at the electronic ground state.
